# What every neurosurgeon should know about the Web 2.0

**DOI:** 10.4103/2152-7806.64965

**Published:** 2010-06-30

**Authors:** Pieter L. Kubben

**Affiliations:** Department of Neurosurgery, Maastricht University Medical Center, The Netherlands

Last month you could read an introduction to “Neurosurgery 2.0”. It has been defined as “an online platform to provide Web 2.0 services for neuroscience in general, and neurosurgery in particular.” This month there will be explicit focus on what Web 2.0 is and what it has to offer you.

## FROM WEB 1.0 TO WEB 2.0

In 1991 the world wide web (WWW) has been invented by Sir Tim Berners-Lee while working for the European Organization for Nuclear Research (CERN) in Switzerland.[7] The classical WWW is a combination of network protocols and hypertext; it allows users to navigate between pages by following weblinks (also named hyperlinks). These pages contain static information, meaning that content (mainly text) was published by the webmaster and did not offer any user interaction. Gradually pages became more interactive and dynamic, meaning that user input is used to decide what content is displayed. Still, the webmaster owns the content of the page.

In 2004 the concept of Web 2.0 was introduced by Tim O'Reilly *et al*., which focused on the online paradigm shift from “publishing” towards “participating.”[9] Web content is not owned by the webmaster anymore. Instead, the website is an environment (a so-called “web service”) where users add value to the website while using it. Basically users are trusted as “co-developers” to “harness collective intelligence.” Meanwhile, content should be accessible from a broad range of devices (including mobile) using interactive user interfaces.

## COLLECTIVE INTELLIGENCE

If Web 2.0 is about new methods to acquire and share data to harness collective intelligence, the question is, what intelligence? In this context it refers to everything users want to share, for example: text, photos, videos, weblinks, keywords for sorting content but also even social networks. The new Web 2.0 horizons lead to cover articles in journals like BusinessWeek (“The Power of Us”) and Time (“Person of the Year: YOU”).[8,10] Medical journals have shown their interest as well. An editorial in the *British Medical Journal* describes four futures of medical publishing that all depend heavily on electronic communication.[2] An editorial in “Nature Medicine” also discusses the increasing value of Web 2.0.[1] It provocatively states that “in the future, Nature Medicine will be famous to 15 people,” suggesting that the printed journal may cease to exist. Web 2.0 can take the lead, and in the last few years, scientific papers on this topic are getting published more frequently.

The list of Web 2.0 services that are currently available is overwhelming. This editorial will focus on the most important ones that are relevant for your practice, your network and your career.

## WIKI

The word *wiki* comes from Hawaii and means “quick.” An online wiki is a website that “allows the easy creation and editing of any number of interlinked web pages via a web browser.”[5] Typically anyone can edit the web pages, although modifications that require approval are in use. The most famous example is Wikipedia.org, which is a free online encyclopedia. A study published in “Nature” demonstrated that the accuracy of Wikipedia and that of Encyclopedia Britannica are comparable, favoring online collaboration as a useful tool to harness collective intelligence.[6] A wiki of interest for neurosurgery and neuroscience is available on the website of the Congress of Neurological Surgeons (wiki.cns.org).

## YOUTUBE

YouTube is an online video-sharing service. Everyone can create a free account and post videos up to 10 minutes in length. Besides, users can comment on other videos and share videos on social networks. Based on watched videos, the website offers suggestions on what other videos you may like. Although YouTube is the most well-known online video-sharing service, there are several alternatives. For “Surgical Neurology International” (SNI), we chose Vimeo because they do not set a time limit on video length. Especially, surgical videos or lectures can be longer than 10 minutes, which makes them unsuitable for YouTube. You can find several movies in the Multimedia menu on the SNI website. There is a category “How I Do It” by Dr. Atos de Sousa from Brazil, and there is the UCLA 100 Lecture Series.

Watching online movies on a mobile device can be troublesome. Most web-based video players use the Adobe Flash player, which is not yet available for all devices and will not be available on the iPhone / iPad. In the future, most websites will switch to a different approach (called HTML5). Until then, users need a mobile version of the video player. YouTube is widely supported on mobile devices, but there is an alternative: podcasts.

## PODCASTS

A podcast is “a series of digital media files (either audio or video) that are released episodically and often downloaded through web syndication” (syndication will be explained in the next paragraph).[4] Originally this was called “webcast,” but it was changed to podcast due to the iPod's popularity for using them. Simply said, a podcast allows you to subscribe to a media source and receive updates automatically. The downloaded media can be accessed without internet access, and many mobile devices nowadays offer support for (video-)podcasts as well. The UCLA 100 Lectures Series are available as podcasts, and the “How I Do It” category will be available as podcasts shortly.

## RSS FEEDS

RSS stands for “really simple syndication” and is a method to subscribe to web pages comparable with the method that podcasts use to let you subscribe to media files. If there is new content available on the website (both text and other media), the user can receive those updates in an RSS reader. Subscribing to a website means subscribing to the (RSS) feed of that website. A feed is nothing more than a protocol that contains some basic information on the website and all its updates. Suppose you have 10 or even 20 websites that you like to check regularly; if all those sites offer RSS, you can subscribe to these sites and view all updates at one place. This place can be either a web-based RSS reader or software you install on your computer. To some extent, RSS can be compared to e-mail; but in RSS, the messages are sent by websites you subscribe to instead of other people that have your address.

An increasing number of scientific journals use RSS feeds to alert its readers when new articles are available. On the SNI website, you can subscribe separately to the RSS feed for new articles (in the menu, click Articles > Free Subscriptions > RSS feed) and to the Posts. Technically speaking, what we call “Posts” on SNI is actually blog, and using RSS feeds in blogs is very common.

## BLOGS

A blog (short for “weblog”) is a specific type of website where typically the webmaster regularly posts content (text, multimedia), and where users can react with comments. Posted topics are usually displayed in reverse chronological order. If you want to follow a blog, it can be convenient to use its RSS feed. Instead of regularly visiting the website to see if new content has been added, you will be notified automatically. Blogs are available on many topics, including neurosurgery and neuroscience. SNI uses a blog in the Posts category, where “Case of the Month” and “Residents' Corner” are two available categories, but there will be some more in the next few months.

## TWITTER

Twitter is the most used web service for “microblogging.” Users can post short messages (called “tweets”) with a maximum of 140 characters. It is successful due to its brevity: you can easily scan quite a lot of tweets in a short time. Many tweets contain links to websites (like interesting topics, blog posts or videos), and you can click on them if the topic interests you. You can not only “follow” other people's tweets but also certain topics of your interest. And you can share (“retweet”) other people's posts with a reference to their name. This creates a sort of microcosm with a wealth of information stored on a central place: Twitter.com.

SNI uses Twitter in two ways. First, if you subscribe to the SNI tweets, you will receive links to all updates in the journal automatically (both articles and posts). Second, other interesting tweets and links will be (re-)tweeted for you to stay up-to-date. On the SNI website, click on “Neurosurgery 2.0 > Social Networks > Twitter” to read our tweets and subscribe to them.

Google Buzz is comparable to Twitter, but it does not have the length limit of 140 characters. It's fairly new and we are using it to test its added value. You can find it on the website in the same Social Networks menu.

## SOCIAL NETWORKS

Online communities offer new methods for connecting to other people and collaborating. Twitter was mentioned separately, because it offers great opportunities to stay up-to-date easily and quickly. Other communities are Facebook and LinkedIn.

A quote on Twitter says: “Facebook is for talking to the people you went to school with. Twitter is for talking to the people you wished you went to school with.”[3] In essence, this summarizes the difference between both networks: Facebook is more informal, Twitter is more functional. As SNI wishes to be both, you can find our Facebook page under the Social Networks section as mentioned before.

LinkedIn is a professional community where you can connect to your colleagues, earlier colleagues and other professional relatives. You can browse through networks of people you are connected with, which may help you to get new connections as well. Further, you can become a member of groups where you can post topics to discuss and join discussions started by other people. You will find the LinkedIn SNI group in the Social Networks section.

## NOW WHAT?

The intention of this editorial is to familiarize you with Web 2.0 and what sort of web services it has to offer you. If you are interested in learning more on a certain topic, you can visit the SNI website and go to Neurosurgery 2.0 > Instructions. This is a new section that not only shortly repeats the quick introduction on each topic but also provides you with the information and weblinks you need to get started. How do you subscribe to RSS feeds? How do you start using Twitter? And more importantly, this section guides you on vital questions such as these: What are the useful blogs for you to follow? What tweets would be worth your time? Of course, this is highly individual, but *this* is the place to get started. And to participate ― if you have questions, comments or additions, I would like to hear them!


Pieter Kubben
Neurosurgery 2.0 Editor
Surgical Neurology International

